# Design and Experimental Evaluation of an Odor Sensing Method for a Pocket-Sized Quadcopter

**DOI:** 10.3390/s18113720

**Published:** 2018-11-01

**Authors:** Shunsuke Shigaki, Muhamad Rausyan Fikri, Daisuke Kurabayashi

**Affiliations:** 1 Division of Systems Research, Yokohama National University, 79-5 Tokiwadai, Hodogaya-ku, Yokohama 240-8501, Japan; 2 Department of Systems and Control Engineering, Tokyo Institute of Technology, 2-12-1 Ookayama, Meguro-ku, Tokyo 152-8552, Japan; mrfikri@irs.ctrl.titech.ac.jp (M.R.F.); dkura@irs.ctrl.titech.ac.jp (D.K.)

**Keywords:** chemical plume tracing, pocket-sized quadcopter, aero-olfactory effect, particle image velocimetry

## Abstract

In this study, we design and verify an intake system using the wake of a pocket-sized quadcopter for the chemical plume tracing (CPT) problem. Solving CPT represents an important technique in the field of engineering because it can be used to perform rescue operations at the time of a disaster and to identify sources of harmful substances. An appropriate intake of air when sensing odors plays an important role in performing CPT. Hence, we used the air flow generated by a quadcopter itself to intake chemical particles into two alcohol sensors. By experimental evaluation, we verified that the quadcopter wake intake method has good directivity and can be used to realize CPT. Concretely, even at various odor source heights, the quadcopter had a three-dimensional CPT success rate of at least 70%. These results imply that, although a further development of three-dimensional CPT is necessary in order to conduct it in unknown and cluttered environments, the intake method proposed in this paper enables a pocket-sized quadcopter to perform three-dimensional CPT.

## 1. Introduction

This article presents how to sense an odor with a pocket-sized quadcopter. The pocket-sized quadcopter is small and has excellent mobility. For the above reasons, researchers have focused their attention on how to control the flight of pocket-sized quadcopters using an onboard camera in order to enter through gaps and move around collapsed buildings at disaster sites [1,2,3,4]. Hence, it is expected that the feasibility and practicality of using pocket-sized quadcopters at disaster sites will be further improved by equipping them with the ability to search for odor sources. However, there is not enough discussion on the mechanism for sensing odors with a pocket-sized quadcopter because it is not clear how the odor distribution around the quadcopter changes due to the rotation of its own propellers. In summary, it is possible that a drone can be used for chemical plume tracing (CPT) as an application if we can enable said drone with an odor-sensing mechanism.

Chemical plume tracing (CPT) represents an important issue in the field of engineering [5]. The literature [6] defines CPT as the navigation of a robot system in response to real-time sensor information so as to locate a plume, trace it toward its source, and identify its source location. This search ability can be used for several humanitarian purposes, such as rescuing victims (e.g., by sensing androstenone [7]) when a disaster occurs and searching for an object in a hazardous area that contains toxic chemicals (e.g., by sensing thionyl chloride [8]). Thus, an effective CPT solution with engineering value is required.

Generally, there is no concentration gradient to use to orient towards the odor source [9]. Insects search odor sources using a plume structure [9,10,11]. Therefore, it is important to acquire a plume structure. However, plume structures change in real-time depending on environmental factors such as airflow direction and obstacles [12]. Therefore, CPT is categorized as an engineering challenge.

In previous studies, two-dimensional ground mobile robots that use wheels were developed to solve the CPT problem [13,14,15,16,17,18]. However, the three-dimensional diffusion of chemicals causes CPT robots to lose information related to the chemicals, unless they can follow it in a three-dimensional manner. Thus, some researchers attempted to solve the CPT problem using three-dimensional movable unmanned aerial vehicles (UAVs) [19,20,21,22].

One study [19,20] used a large UAV (AR100-B, AirRobot, Arnsberg, Germany), with a diameter of approximately 1 m, and employed a search algorithm that predominantly used information from wind direction. At a disaster site, it is necessary not only to search wide areas, but also to enter narrow places and search for odor sources. The size of the doorway of a typical building approximately corresponds to 1 m [23], and thus it is difficult to enter the building and explore the chemical source using large quadcopters. Moreover, a UAV is unable to measure natural wind direction accurately because of the wind flow created by its own propellers [19,24]. In an attempt to overcome this problem, previous works executed odor source search while estimating the odor direction [19,20,21]. However, an accurate estimation of wind direction can only by achieved in simulations. Hence, in real conditions, inaccurate estimations of the wind direction lead to a decreased CPT performance. In an environment with many obstacles, such as a forest, the search algorithm using information about the wind direction is not effective, because the direction of an odor does not completely match the direction of the wind [25]. Hence, the UAV may not search for an odor source in a windless and narrow space in a building.

When wind information cannot be used, it is important to determine from which direction the odor is detected. For this reason, it is necessary to install a mechanism that can give directivity in odor detection to the pocket-sized quadcopter. However, in the case of a pocket-sized quadcopter, it is not possible to install an additional actuator on the quadcopter due to the payload limitation.

Hence, we can summarize the aim of this paper as: to design and evaluate a mechanism that can sense odor without an additional actuator. Our approach to fulfill this aim is to use the aero-olfactory effect of the quadcopter [26] in order to sense chemicals. This aero-olfactory effect is called a “wake” in the literature [22], so we use the word “wake” in this paper. The “wake” is a phenomenon in which a strong odor distribution occurs around the quadcopter due to the influence of the rotation of the propellers [22]. Also, we conduct two types of experiments to evaluate the basic performance of the sensing mechanism using wake. After that, we investigate whether CPT can be performed by the wake-based sensing mechanism. Finally, we carry out CPT experiments in a free space as an evaluation of the performance of wake-based sensing.

## 2. Problem Statement

First, we define the quadcopter class. In the UAV industry, and especially for quadcopters, it is not possible to classify the quadcopter class accurately, because a global standard for the classification does not exist. Therefore, in this study, we define the quadcopter class as shown in Table 1 by referring to [23]. Specifically, “Indoor” in Table 1 indicates whether the quadcopter can enter and safely fly inside a building. The meaning of the symbols “◯”, “△” and “×” in the column “Indoor” are shown below.

- ◯: The quadcopter can pass through any door, and it can safely fly inside a building.
- △: The quadcopter cannot pass through some doors, and sometimes, it is difficult for it to fly inside a building.
- ×: The quadcopter cannot pass through any door and fly safely inside a building.

In this study, we selected a pocket-sized quadcopter because the aim includes searching for an odor source in a narrow space at the time of a disaster. In addition, we selected a pocket-sized class from the viewpoint of the safety of the victims at a disaster site and the cooperative work of the quadcopters. As the size of the quadcopter increases, the price per unit rises, and the risk of injury due to the rotation of the propellers increases. Therefore, we assume that using a pocket-sized quadcopter is better than using larger ones for CPT at disaster sites.

We employed the Airborne Night Blaze (Parrot SA, Paris, France) as the pocket-sized quadcopter platform, because the flight of the quadcopter itself is controlled by an installed flight controller, and we can easily operate the quadcopter by simply sending a behavioral command via Bluetooth Low Energy (BLE). The Airborne Night Blaze includes four 65 mm-diameter propellers, and the size of the quadcopter is approximately 150 × 150 × 40 mm. The systems were developed on a small platform, and thus, it is necessary to consider several parts, mainly with respect to the payload in mounting important devices that control the pocket-sized quadcopter to complete CPT missions. We can implement only the minimum number and type of sensors required for CPT on the quadcopter because the maximum loading capacity is only 40 g. The pocket-sized quadcopter can fly for about 9 min without loads and for about 6 min when carrying loads. From the perspective described above, we should select an algorithm that is suitable for the system. In this study, we used ethanol as the target chemical from the viewpoint of experimental safety and ease of availability. For this reason, we detected the presence or absence of the chemical in air using an alcohol sensor. We aim to develop a pocket-sized quadcopter CPT system by considering the following items.

1. Design a lightweight and compact system with a weight of 40 g or less to detect the presence/absence of a chemical (ethanol).
2. Select and implement an algorithm that can realize three-dimensional CPT in real time.
3. Design and verify an intake system that does not require additional actuators. Here, the intake system implies how to intake air into the alcohol sensor.

Regarding the CPT algorithm itself, we draw inspiration from flying insects that use not only olfaction information, but also wind direction information during CPT [27,28,29,30]. Additionally, walking insects (e.g., cockroach) can perform CPT without using bilateral olfactory information [31]. This is because the cockroach can detect particles even with one antenna because said antenna has a very high number of receptors [32]. We decided to use the search algorithm of the silkworm moth *Bombyx mori* that realizes CPT using bilateral olfactory information [33,34] because it is difficult to use wind direction information and equip pocket-sized quadcopters with a large number of sensors, as mentioned previously. Therefore, we equipped the pocket-sized quadcopter with two alcohol sensors. However, we extend the silkworm moth CPT algorithm to three dimensions since the original algorithm of the silkworm moth is limited to movement in two-dimensional planes [33,35]. Furthermore, we adopt the moth-inspired algorithm because it does not need accurate location or odometry since it is not a cognitive algorithm, but a reactive one.

The correct detection of chemicals plays the most important role in completing CPT. This includes appropriately intaking the air to an odor sensor, as well as sampling the chemical from the front of the quadcopter only and not from other directions. This has the effect of preventing the quadcopter from moving in a direction that is not an odor source. A UAV in a previous study introduced an intake system that used an additional fan inside the intake system for airflow control [19,20]. However, in the case of a pocket-sized quadcopter, it is not possible to install an additional actuator on the quadcopter due to the payload limitation. Thus, we focus on the wake of the quadcopter to design the intake system. However, detailed airflow distribution caused by the wake is not known. It is necessary to investigate the airflow distribution around the quadcopter using the wake and to determine the position to install the alcohol sensors.

To evaluate the performance of the intake system using the wake, we performed two key experiments. One is an experiment to verify the odorant detection directivity, and the other is to verify whether it can be used for CPT.

## 3. Construction of a Pocket-Sized Quadcopter System

### 3.1. System Overview

**Figure 1:**
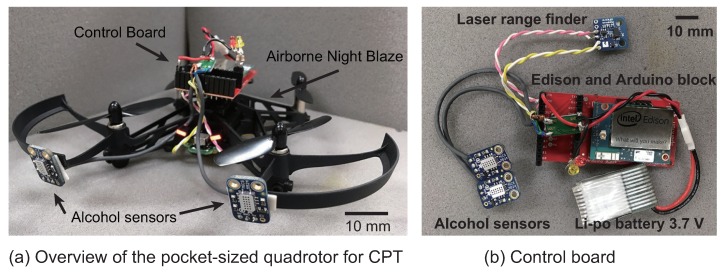
Overview of an autonomous pocket-sized quadcopter system for CPT. The system consists of a control board and a pocket-sized quadcopter.

**Figure 2:**
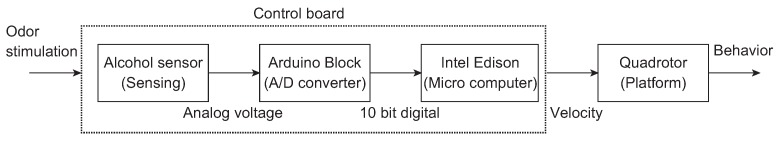
Series of information processing flows ranging from detecting odors to controlling the quadcopter.

Figure 1 shows a schematic diagram of the pocket-sized quadcopter system intended to search for chemical odor sources. The entire system consists of a control board and a pocket-sized quadcopter. Figure 2 shows the flow from the acquisition of environmental data using sensors to controlling the quadcopter with respect to the control board and the quadcopter. In the control board, two alcohol sensors observe the presence or absence of chemical information, and the Intel Edison that plays the role of a CPU determines subsequent search behavior states. In addition, a laser range finder (VL53L0X, STMicroelectronics, Geneva, Switzerland) for measuring the altitude of the quadcopter is attached to the lower part of the quadcopter. The pocket-sized quadcopter controls the position and speed using only an internal controller, and we do not use any external controllers, e.g., motion capture or a camera.

We explain the details of each component in subsequent sections.

### 3.2. Control Board

The role of the control board is to determine the direction of the quadcopter from the values obtained by the alcohol sensors, as shown by the dotted line in Figure 2. Figure 1b shows the constructed control board. We use the control board installed on the pocket-sized quadcopter, as shown in Figure 1a. The control board consists of two alcohol sensors, a laser range finder, an A/D converter, a microcomputer and a power supply circuit.

We connected two MiCS5524 gas sensors (alcohol sensors) to the microcomputer via the A/D converter to acquire the sensor values. In addition, we connected the laser range finder to the microcomputer via the I^2^C (Inter-Integrated Circuit) interface and measured the altitude of the quadcopter every step. We applied a moving average low pass filter of 5 Hz to remove the high-frequency noise of the laser range finder sensor value. We used an Intel Edison (Intel, Santa Clara, CA, USA) and an Intel Edison Block-Arduino (SparkFun, Boulder, CO, USA), which are extremely lightweight and compact, for the microcomputer and A/D converter, respectively. The Intel Edison Block-Arduino converted the analog value of the sensor into a 10-bit digital signal and sent it to the Intel Edison. The Intel Edison is equipped with a dual-core Intel Atom processor at 500 MHz, with Yocto Linux installed on it. The Intel Edison executed a series of processes, from the acquisition of the alcohol sensor values to commanding the behavior state of the quadcopter. The series of processing codes was written using Node.js, which corresponds to a server-side JavaScript. The processing program for the control board runs at a 30-Hz control cycle. We used the “node-rolling-spider” library [36] to control the quadcopter. This Node.js library is necessary for sending the direction commands from the script running on the control board into the internal flight controller of the quadcopter via BLE. The total weight of the control board was approximately 30.9 g, and it included the battery that drives the sensors. This value was less than the maximum payload of the pocket-sized quadcopter, that is 40 g. Consequently, we successfully developed a lightweight system to detect the presence/absence of ethanol.

### 3.3. System Identification of an Alcohol Sensor

In this study, we used ethanol as the chemical, given the requirements of safety and availability. Subsequently, we used a MiCS5524 gas sensor breakout (Adafruit, New York, NY, USA) [37] that displays high sensitivity and a fast response to ethanol. The MiCS5524 is a compact MOS sensor, and it can detect ethanol, carbon monoxide, hydrogen, ammonia and methane [37]. Detection of chemicals is achieved by measuring the sensing resistance of the sensor. The resistance decreases in the presence of a chemical. Moreover, the sensor has a heater that overheats the adhered chemical; therefore, the output value of the MiCS5524 sensor varies depending on ambient temperature and wind speed [38,39]. The performance of the sensor for CPT was considered successful if the quadcopter accurately estimated a true chemical input. Given the characteristics of the MiCS5524 gas sensor breakout, it is not possible for the microcomputer of the quadcopter to determine a true input by simply setting a threshold value for the sensor output, because the base voltage increases when the alcohol sensor detects alcohol with a high frequency. Thus, we applied an inverse sensor model to the sensor output value to estimate the presence or absence of alcohol in the atmosphere [40]. We used an autoregressive with exogenous input (ARX) model as the inverse model [41,42], because the ARX model represents a wide class of nonlinear systems with a low number of parameters [43].

We used the ARX model of Equations (1) and (2) to estimate ethanol detection.

(1) $$\hat{u}\left(k\right)=b_{0}\dot{y}\left(k\right)+b_{1}\dot{y}\left(k-1\right)-a_{1}\hat{u}\left(k-1\right)-a_{2}\hat{u}\left(k-2\right)$$

(2) $${\hat{u}}_{bin}\left(k\right)=\left\{\begin{array}{cc} \mathrm{Detect} & \left(\hat{u}\left(k\right)\geq \mathrm{Threshold}\right)\\ \mathrm{Not}\;\mathrm{detect} & \left(\mathrm{otherwise}\right) \end{array}\right.$$

Here, $\hat{u}\left(k\right)$ represents the estimated value of the input at the *k*-th step, ${\hat{u}}_{bin}\left(k\right)$ is the estimated value of the input at the *k*-th step represented by a binary value and $\dot{y}\left(k\right)$ (V/s) represents the time differentiation of the sensor output voltage at the *k*-th step. $a_{1}$, $a_{2}$, $b_{0}$, $b_{1}$ are coefficients of the ARX model. To estimate the unknown coefficients, we provided an M-sequence of ethanol stimuli to the MiCS5524 gas sensor. As an experimental setup for estimating the coefficients, we put a discharge port at a position 10 mm in front of the MiCS5524 gas sensor to ensure that the ethanol reaches the sensor. The ethanol discharge port is made as a glass tube with a diameter of 5 mm, and we inserted a filter paper (10 × 10 mm^2^) with 10% ethanol into the glass tube. The ethanol stimulator consists of an air compressor (MAS-1, AS ONE, Osaka, Japan), three gas washing bottles (absorbent cotton, activated carbon and distilled water), a flow meter (RK1710-AIR, KOFLOC, Kyoto, Japan) and a solenoid valve (VT307, SMC Corporation, Tokyo, Japan). The air expelled from the air compressor is passed through absorbent cotton, activated carbon and distilled water, adjusted to a constant flow rate of 1.0 L/min by the flowmeter, and presented to the MiCS5524 gas sensor. The stimulation timing to the MiCS5524 gas sensor is controlled by changing the state of the solenoid valve. We used the least squares method, which is the most representative method to determine the parameter value for the ARX model [41,42]. We used the System Identification Toolbox of MATLAB [42] to select the coefficients of the least squares method for the ARX model. Specifically, the ARX model coefficients vector Θ is estimated by Equation (3). Here, J and Y are the regressor matrix and the measured sensor output, respectively.

(3) $$\begin{array}{ccc} \left(J^{T}J\right)\Theta & = & J^{T}Y\\ \Theta & = & {\left(J^{T}J\right)}^{-1}J^{T}Y \end{array}$$

Table 2 shows the estimation results of these coefficients.

**Figure 3:**
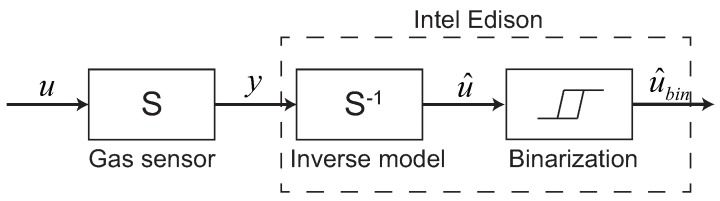
The flow of odor information processing using the ARX inverse model.

Figure 3 shows a flow of odor information processing using the ARX model. Here, *u*, *y*, $\hat{u}$ and ${\hat{u}}_{bin}$ indicate ethanol input, gas sensor output, estimated input using ARX inverse model and binarized estimated input, respectively. As shown in Figure 3, we implemented the ARX model and binarization in the Intel Edison.

**Figure 4:**
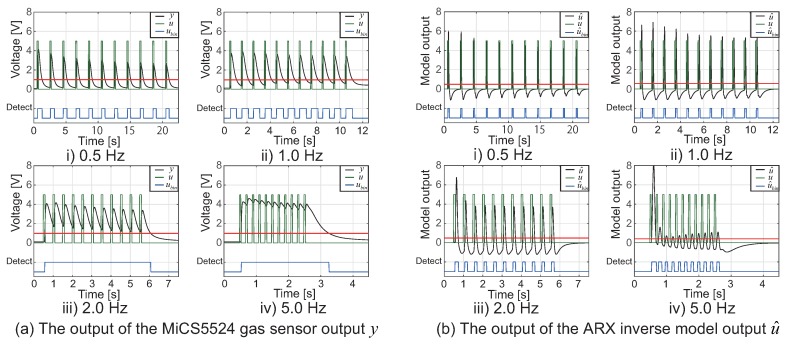
The MiCS5524 gas sensor response and ARX inverse model result for periodic odor stimuli. The green line represents the opening/closing timing of the solenoid valve (stimulation supply timing), and the blue line represents the result of binarizing *y* or $\hat{u}$ with the threshold value (red line). The black lines of (**a**,**b**) indicate MiCS5524 gas sensor output *y* (V) and estimated input $\hat{u}$ calculated by using ARX inverse model, respectively. We set thresholds for *y* and $\hat{u}$ to “1” and “0.5”, respectively.

To verify the effectiveness of applying the ARX model to the MiCS5524 gas sensor, we carried out an experiment to give odor stimuli with four different frequencies (0.5, 1.0, 2.0, 5.0 Hz) to it. Moreover, we used the same environment as the one in the experiment that provided the M-sequence of ethanol stimuli. Figure 4a shows the MiCS5524 gas sensor response, *y*, when we provided it with periodic odor stimuli. The results of applying the ARX model to the gas sensor response *y* are shown in Figure 4b.

The green line *u* in Figure 4 represents the timing at which the solenoid valve is opened. This corresponds to the timing when the ethanol stimulus is given to the MiCS5524 gas sensor. In addition, the blue line shown in Figure 4 indicates the result of binarizing the sensor response *y* with the threshold value (red line). We set thresholds for *y* and $\hat{u}$ to “1” and “0.5”, respectively. We determined these threshold values empirically. These thresholds were used in all the experiments in this paper. As shown in Figure 4a, the recovery time of the MiCS5524 gas sensor is slow. For this reason, it is difficult to distinguish the stimulation timing and to simply set the threshold value for the MiCS5524 gas sensor *y* because the base voltage rises when ethanol is detected with a high frequency. Conversely, by applying the ARX inverse model to the gas sensor output, *y* shown in Figure 4b, we can discriminate the stimulation timing even with high-frequency stimuli. As a result, we adopted the ARX inverse model for the pocket-sized quadcopter because it offers sufficient performance for the accurate detection of odors in the atmosphere.

### 3.4. Search Algorithm

**Figure 5:**
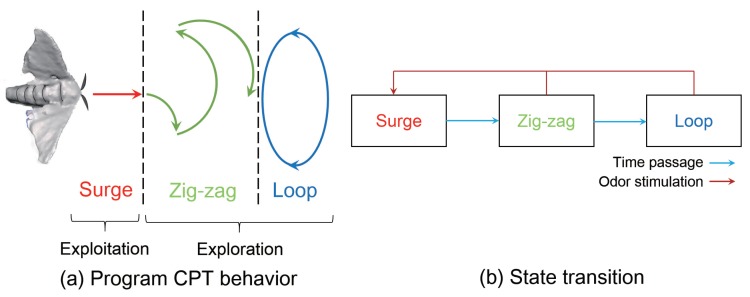
CPT behavior of a male silkworm moth.

We used a search algorithm that imitates the behavior of an animal that searches for an odor source and installed it in the pocket-sized quadcopter. In this study, we focused on the CPT behavior of an adult male silkworm moth, *Bombyx mori*. The adult male silkworm moth elicits a stereotypical behavior pattern, as shown in Figure 5, when it receives the sex pheromone of a female [33]. A male silkworm moth reaches a female using odor information obtained while walking, because it cannot fly, despite possessing wings [44]. The CPT behavior of the male silkworm moth consists of the following three patterns, as shown in Figure 5a: “surge”, “zig-zag” and “loop”. “Surge” denotes exploitation behavior (going straight), while “zig-zag” and “loop” denote exploration behaviors (rotational motion). As shown in Figure 5b, there are two state transitions of conditions in the CPT behavior. A state transition (Figure 5b) occurs when the silkworm moth detects a stimulus (red line), and the other state transition occurs over time (light blue line). Thus, the surge state occurs when the moth encounters a pheromone stimulus.

**Figure 6:**
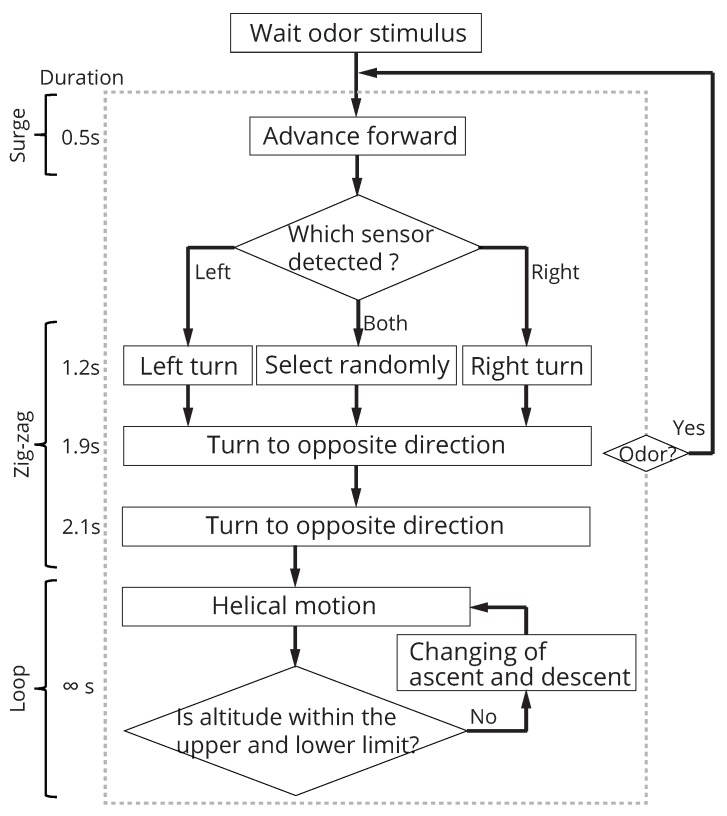
The flowchart of the three-dimensional silkworm-moth-inspired CPT algorithm.

An odor source was searched by implementing this simple behavior pattern on our pocket-sized quadcopter. However, we need to extend the silkworm moth-inspired algorithm to three dimensions because the silkworm moth searches on a two-dimensional plane. Figure 6 shows the flowchart of the silkworm moth-inspired algorithm extended to three dimensions. This algorithm also selects the same three behavioral states as the silkworm moth: surge, zig-zag and loop. The duration of each behavior state is shown on the left side of Figure 6. We set the same duration for each state in coherence with a behavioral experiment of a previous study [33].

As shown in Figure 6, the initial turn direction at the zig-zag state is determined according to the stimulated antenna (left of right) [33,34]. When both antennae respond, the initial turn direction is randomly selected [34]. Here, the algorithm uses information on the detection timing rather than the concentration of the chemical. In the surge and zigzag states, the quadcopter executes the search behavior while maintaining its altitude at the time of detecting an odor using the sensor. When transitioning to the loop state, the quadcopter searches for the odor three-dimensionally while vertically changing its altitude by performing a helical motion. We set the altitude range of the loop state to range from 0.6–1.4 m. In addition, we set the speed of the height direction to 0.04 m/s so that the quadcopter searches between the lower limit and upper limit of the height direction in 20 s. We measured the altitude using a range finder mounted on the bottom of the quadcopter. The translational and angular velocities of the quadcopter were set as 0.25 m/s and 1 rad/s, respectively.

## 4. Determination of Sensor Arrangement

In this section, we determine the position and direction of the alcohol sensors. To assess the optimal sensor position, we used the wake of a pocket-sized quadcopter for intaking air into the alcohol sensors. However, we did not know where to position the alcohol sensors. Therefore, we used particle image velocimetry (PIV) [45] to visualize the airflow generated by the wake around the quadcopter. The PIV is a method of mixing particles that follow a fluid into a flow field to obtain displacement vectors of particles at a time after the consecutive capture of visualized images and to estimate the velocity vector.

We selected sprinkle water and propylene glycol mixture particles whose diameters were less than 10 µm (PS-2005, KATOKOKEN Co., LTD, Kanagawa, Japan) for the PIV experiment, to ensure particle tracking performance relative to the predicted airflow velocity of the quadcopter (see the Appendix for the particle-tracking performance). We measured the particles with a high-speed camera (k4, KATOKOKEN Co., LTD, Kanagawa, Japan) to calculate the velocity vector. We set the frame rate and the image size of the high-speed camera at 300 fps and 800 × 600, respectively.

**Figure 7:**
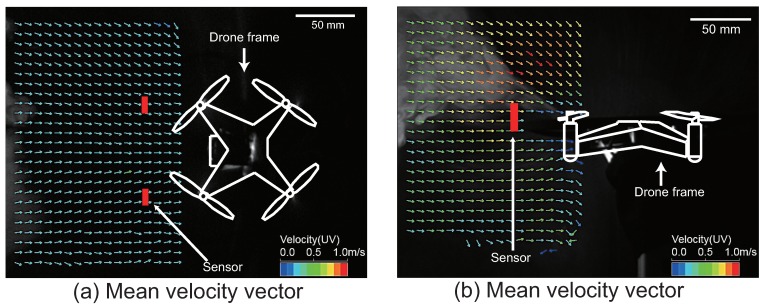
Quadcopter top and lateral view of the air velocity vector by the PIV measurement. The direction of the arrow represents the direction of the airflow, and the difference in color represents the speed. The PIV image corresponds to the average vector per second.

Figure 7a,b shows the average vector for 1 s during the hovering state. We measured the area in front of the quadcopter (230 mm × 150 mm). We calculated the particle velocity using the commonly-used direct cross-correlation method [46]. Figure 7a,b visualizes the airflow around the quadcopter when viewed from the lateral and upper sides of the quadcopter. The white lines in Figure 7a,b represent the quadcopter frame. The direction and color of the arrows represent the direction and speed of the air flow, respectively. The results of the PIV indicate that the quadcopter itself initially captures air from the obliquely upward forward direction using the wake. The operating principle of the alcohol sensor is that chemicals adhere to the heater inside the sensor and cause a change in the electrical conductivity due to catalytic reaction [37]. Consequently, the sensor’s heater cools down where the wind speed is high, and there is a possibility that the sensitivity of the sensor drops [38]. Therefore, we installed alcohol sensors at the positions indicated by the red rectangle in Figure 7, because the red rectangle’s position permits intake of air by the sensor at half the maximum wind speed. The arrangement of the alcohol sensor in the aforementioned places makes it possible to use the wake for the intake of an appropriate amount of air to the sensor.

**Figure 8:**
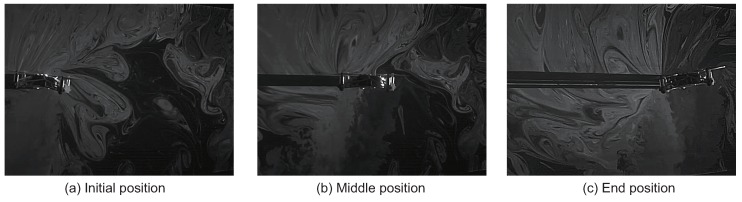
Snapshot of the quadcopter moving through the particles for the PIV analysis. We sent the stop command in the (**b**) position (see Supplemental Video S1a).

**Figure 9:**
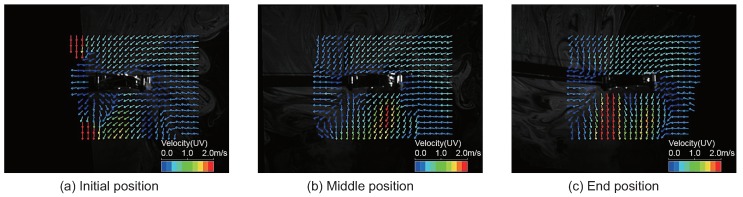
Snapshot of the PIV analysis during the quadcopter flight. We sent the stop command in the (**b**) position. The arrow of the snapshot represents the average velocity vector for 10 ms (see Supplemental Video S1b).

Next, we analyzed the airflow around the quadcopter during flight (see Supplemental Video S1a). In this experiment, we set the frame rate and the image size of the high-speed camera at 500 fps and 640 × 480, respectively. The quadcopter moved approximately 300 mm forward, and we sent a stop command to the quadcopter when it passed 300 mm, as shown in Figure 8. Figure 8a–c represents snapshots of the initial position, the middle position and the end position, respectively. In Figure 8b, the quadcopter began to change its altitude in preparation for stopping. Finally, the altitude of the quadcopter completely changed due to the stop motion, as shown in Figure 8c. Even in these series of movements, we verified that air intake was stable in the place where the alcohol sensor was arranged. To create a fixed coordinate for the flying quadcopter, we performed an affine transformation in the video, as shown in Figure 8. Thereafter, we performed PIV analysis on the converted video (see Supplemental Video S1b). Figure 9 shows a snapshot of the result of the PIV analysis. The arrows of the snapshot represent the average velocity vector for 10 ms. Figure 9a–c represents the PIV result for the initial position, middle position and end position, respectively. It was found that the quadcopter intakes air from the same direction while moving as while hovering.

Therefore, we assumed that the pocket-sized quadcopter can achieve CPT using alcohol sensors installed at the front side of the quadcopter.

## 5. Directivity Experiment of Odor Detection

In this experiment, we verify whether the quadcopter had the needed odor directivity using the determined sensor arrangement. The odorant directivity is important in using the silkworm moth CPT algorithm because it employs stereo olfaction information [34]. In other words, it is important that the detection rate be high when the quadcopter faces the direction of the odor source, and low when it faces the opposite direction. Hence, we conducted two experiments to verify the performance of the directivity of odor detection using the determined sensor arrangement. In the first experiment (Experiment 1), we conducted an experiment to investigate the basic performance of the odorant directivity. In the second experiment (Experiment 2), we investigated the change in the detection rate when the distance and height from the odor source are changed in free space. Through these two experiments, we evaluated whether or not the pocket-sized quadcopter CPT system possesses the odor detection capability necessary for three-dimensional CPT. We describe these two experiments in the following sections.

### 5.1. Experiment 1: Experimental Design

**Figure 10:**
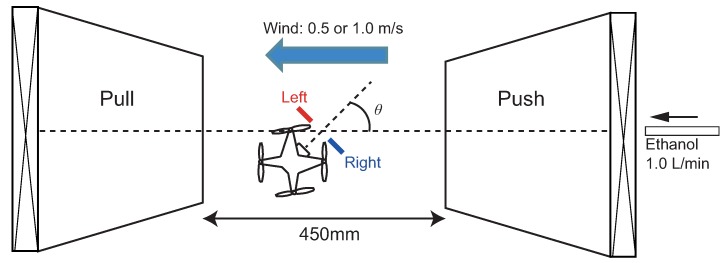
Experiment setup for investigating the odorant detection directivity using the push-pull ventilation system.

In this experiment, we investigated the fundamental performance of the odorant detection directivity using the wake of the quadcopter. We conducted the experiment in a uniform airflow environment. We generated uniform airflow using a push-pull ventilation system [47]. Using this system, we could supply uniform airflow without covering the workspace with partitions, ensuring an effective use of space. As shown in Figure 10, we set the quadcopter equipped with two alcohol sensors at the center of the push-pull ventilation system. We fixed the quadcopter with a monopod to prevent its flight. Moreover, we installed an ethanol discharge port behind the fan on the push side. The ethanol discharge port is made of a glass pipe with a diameter of 5 mm, and we inserted a filter paper (10 × 10 $mm^{2}$) with 99.5% ethanol (50 µL) into the glass pipe. We set the distance between the fan of the push side and the exhaust port to 10 mm. We used a solenoid valve to release puffs of ethanol. We released puffs at a rate of approximately 1.0 L/min.

As we showed in the results of the PIV experiment, the wind speed generated by the floating airflow of the quadcopter is about 1.0 m/s; therefore, we set the wind speed of the push-pull ventilation system to two conditions: 0.5 m/s and 1.0 m/s. In addition, we set the rotation state of the quadcopter’s propeller to two states: hovering and stopped. As shown in Figure 10, we define the upwind direction as 0 rad and conducted the experiment by changing the heading angle of the quadcopter every π/6 rad.

We set the stimulation interval to 2.0 s and presented ten ethanol stimuli to the quadcopter in each experiment (20 ethanol stimuli in total). We conducted the experiment twice per heading angle and calculated the detection rate.

### 5.2. Experiment 1: Result

**Figure 11:**
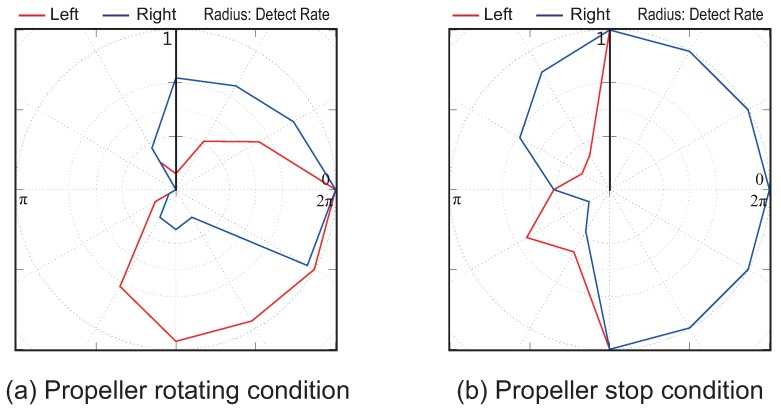
Detection rate results of left and right alcohol sensors at the wind speed of 0.5 m/s. Subfigure (**a**) shows the state in which the propellers of the quadcopter rotate; subfigure (**b**) shows the result for the propellers of the quadcopter stopped.

**Figure 12:**
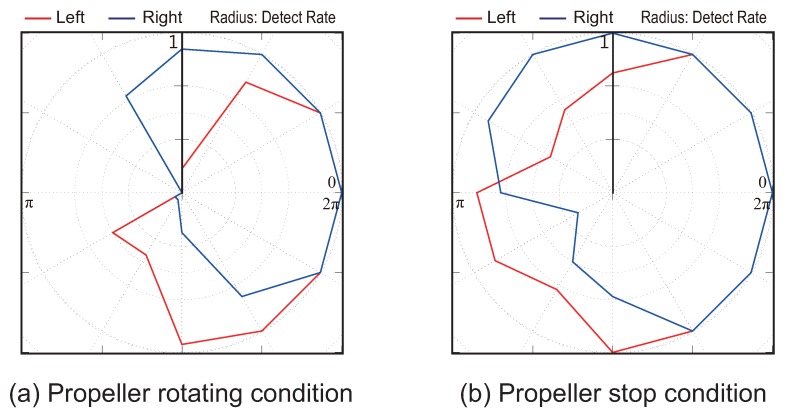
Detection rate results of left and right alcohol sensors at the wind speed of 1.0 m/s. Subfigure (**a**) shows the state in which the propellers of the quadcopter rotate; subfigure (**b**) shows the result for the propellers of the quadcopter stopped.

In this section, we present the results of the experiments to verify the directivity of odor detection using the wake of our quadcopter. Figure 11 and Figure 12 show the results for airflow velocities of 0.5 and 1.0 m/s for the push-pull ventilation system. Moreover, in both figures, (a) and (b) represent the propeller state (rotating or stopped). In addition, the blue and red lines in Figure 11 and Figure 12 indicate the detection rates of the left and right alcohol sensors, respectively. Here, the radius of Figure 11 and Figure 12 represents a detection rate that implies that the sensor responds to all the presented stimuli when the detection rate is “1”.

As shown in Figure 11 and Figure 12, even if the heading angle of the quadcopter faces a direction over 90 degrees upwind, the left and right alcohol sensors almost certainly react. In particular, when the wind speed is high, the detection rate remains high even if the quadcopter faces the opposite direction to the windward direction. However, when the propellers are rotating ((a) of Figure 11 and Figure 12), it was found that the detection rate of the sensor farther from the windward direction is lower at either wind speed conditions. Hence, we found that we can distinguish between left and right odor information using the wake of the quadcopter. In other words, we can discriminate which direction the odor came from because the directivity of odor detection is improved through the use of the quadcopter wake. As a result, we can apply the silkworm moth CPT algorithm that uses stereo olfaction.

Next, we evaluate the odor detection performance in free space.

### 5.3. Experiment 2: Experimental Design

In this experiment, we investigated the effect of the wake of a quadcopter on odor detection in free space. We evaluated the detection performance as the rate of detecting a chemical while changing the distance, height and heading angle between the chemical odor source and a quadcopter equipped with two alcohol sensors. We performed the experiment while keeping the position, height and heading angle of the quadcopter accurately constant using a monopod.

**Figure 13:**
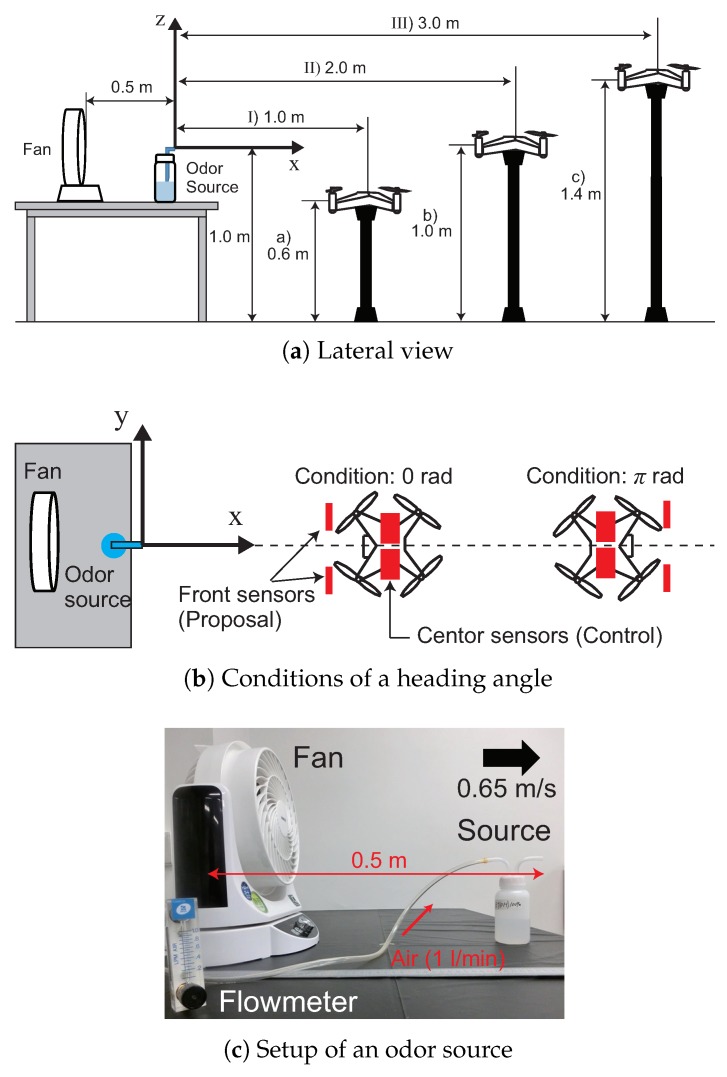
Experimental setup of directional performance.

Figure 13 shows a schematic diagram of the experimental setup. We set a collecting bottle as the odor source at a height of 1 m above the ground. The collecting bottle contained ethanol. As shown in Figure 13c, we installed a fan behind the collecting bottle to disperse ethanol into the air. In this experiment, we controlled the timing of the air injected into the collecting bottle using a solenoid valve. The solenoid valve opened and closed at a cycle of 0.1 Hz (opened: 1 s, closed: 9 s).

We set the distance between the odor source and the pocket-sized quadcopter to (I) 1.0, (II) 2.0 and (III) 3.0 m, and the height to (a) 0.6, (b) 1.0 and (c) 1.4 m, as shown in Figure 13a. As shown in Figure 13b, regarding the heading angle, we conducted experiments for two conditions: the condition while facing the direction of the odor source (condition: 0 rad) and the condition while facing a direction opposite the odor source (condition: π rad). Moreover, we set the position of the quadcopter on the *y*-axis in the line of the odor source. We performed directional experiments according to a total of 18 conditions, while varying the values of distance, height and heading angle.

As shown in Figure 13b, we conducted the experiment on two kinds of sensor arrangements. One is to place the alcohol sensors in front of the quadcopter, determined by the PIV experiment. This sensor arrangement is labeled as “proposal”: in this study. The other is to arrange the alcohol sensors upward at the center of the quadcopter for comparison with the reference [22]. When the sensors were placed at the center of the quadcopter, odor information was acquired from a wide omnidirectional range [22]. We designated this sensor arrangement as “control”. We placed the center sensors at the same height as the front sensors. In this manner, we evaluated the difference in the directivity performance caused by the difference in the sensor placement.

We set the experimental time of one trial as 100 s, and stimuli were released from the odor source 10 times for one trial. We conducted five iterative experiments per condition, and the detection rate, R(n), per condition was calculated using Equation (4),

(4) $$\begin{array}{c} R\left(n\right)=\frac{\sum_{n=1}^{10}{\hat{u}}_{bin}^{L}\left(n\right)/10+\sum_{n=1}^{10}{\hat{u}}_{bin}^{R}\left(n\right)/10}{2} \end{array}$$

where *n* and ${\hat{u}}_{bin}$ represent the number of stimuli and the response of the sensor to the stimulus, respectively. When the sensor responds to the stimulus, ${\hat{u}}_{bin}$ becomes one, and when it does not, ${\hat{u}}_{bin}$ becomes zero. Moreover, the superscripts “*L*” and “*R*” represent the left and right sensors, respectively. We calculated the average and standard deviation of five experiments with respect to this detection rate and used the results for the evaluation.

### 5.4. Experiment 2: Results

**Figure 14:**
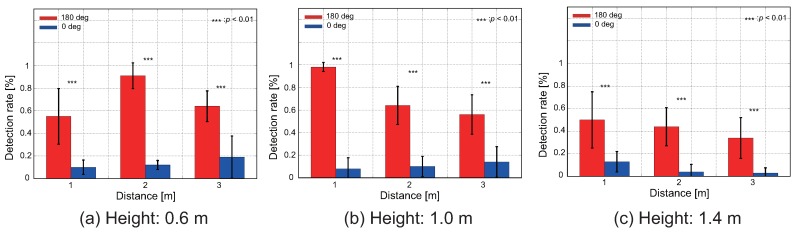
Detection rate of the proposed sensor arrangement. Mean and standard deviations of five iterative experiments are shown. The difference between (**a**–**c**) is the difference in height of the quadcopter. Red and blue bars mean the π rad condition and the 0 rad condition, respectively. We compared differences in detection rate under different heading angle conditions using the Wilcoxon rank sum test (p<0.01).

**Figure 15:**
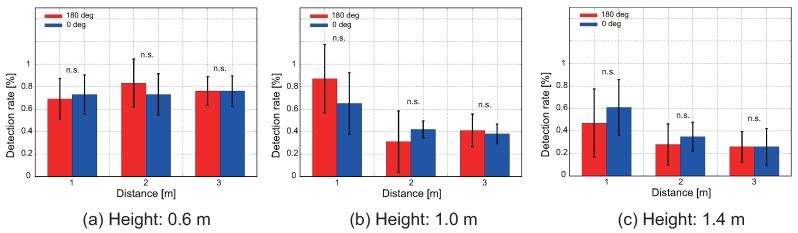
Detection rate of the control sensor arrangement. Mean and standard deviations of five iterative experiments are shown. The difference between (**a**–**c**) is the difference in height of the quadcopter. Red and blue bars mean the π rad condition and the 0 rad condition, respectively. We compared differences in detection rate under different heading angle conditions using the Wilcoxon rank sum test (p<0.01).

We show the results of the verification experiment of the directivity performance of our proposed intake method. Figure 14 and Figure 15 show the proposed condition and the control condition, respectively. The difference between (a–c) is the difference in height of the quadcopter (0.6, 1.0, and 1.4 m). In Figure 14 and Figure 15, the red and blue bars represent the π rad condition and a 0 rad condition, respectively.

As shown in Figure 14, it is clear that the detection rate was higher when the quadcopter faced the odor source direction at any distance and height. We used a Wilcoxon rank sum test to compare the mean values of the detection rate at different heading angle conditions at a significance level of p<0.01. The results showed a significant difference in the detection rate between the 0 rad and π rad conditions. Therefore, it was revealed that the intake method using the wake is effective. When the quadcopter was at the same height as the odor source, the detection rate was always found to be about 60%, even if the distance changed, as shown in Figure 14b. Furthermore, we confirmed that as the distance from the odor source increases, the detection rate increases as the height decreases, because ethanol is heavier than air.

However, under the control sensor arrangement, no significant difference was found in the detection rate, even if the heading angle changes, as shown in Figure 15. Under this condition, as the distance increased, the detection rate also improved if the height was lowered.

Therefore, with this experiment, we confirmed that the intake method in the proposed sensor arrangement is effective for realizing CPT using a pocket-sized quadcopter.

In the next experiment, we investigate the effects of the proposed intake method on CPT performance.

## 6. Three-Dimensional CPT Experiment

### 6.1. Experimental Design

**Figure 16:**
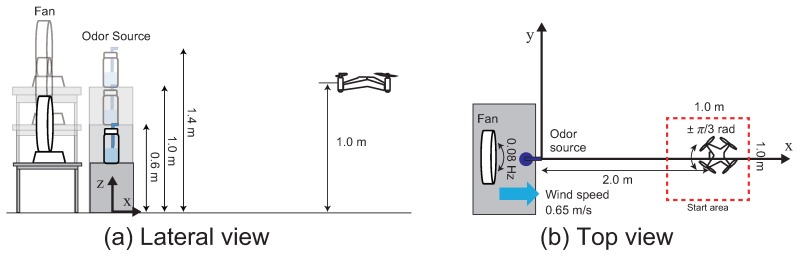
Schematic of the three dimensional CPT experiment field. (**a**) Lateral view; (**b**) top view.

We performed a three-dimensional CPT experiment in an environment where the wind direction was periodically changing. Our experimental room size was 8.0 × 6.0 × 2.5 m^3^. Figure 16 shows a schematic diagram of the experimental field. Figure 16a,b represents the lateral and top views of the CPT experimental environment, respectively. We set the height of the odor source (collection bottle) to three conditions: 0.6, 1.0 or 1.4 m above the ground. We continued to send a constant flow of air (1.0 L/min) to the ethanol in a collecting bottle and dispersed it in the air, as shown in Figure 13c. To promote the diffusion of the ethanol in the +x direction, we placed a fan 0.5 m behind the odor source (−x direction). The wind direction fluctuates periodically because the fan head swings at a constant period (about 0.08 Hz). We set the wind speed generated by the fan to about 0.65 m/s at the measurement point, at a distance of 50 cm from the fan.

We randomly placed the start position of the pocket-sized quadcopter inside the red frame in Figure 16 and conducted the experiments. In addition, with respect to the initial heading angle of the quadcopter, we set it between $\pm \frac{\pi }{3}$ rad. We set the initial altitude of the quadcopter to 1.0 m. The behavior of the pocket-sized quadcopter was recorded using two wide-angle cameras (BSW20KM11BK, BUFFALO INC., Aichi, Japan, 30 fps): one camera was installed on the ceiling (*x*-*y* plane), and the other camera was set on the side of the experiment field (*x*-*z* plane). We synchronized the recording of these cameras at the start of each experiment. The search was considered successful if the quadcopter entered a cylinder centered at the odor source (Equation (5)) within 2 min.

(5) $$\begin{array}{c} \left\{\begin{array}{c} x=0.5cos\left(\theta \right)+x_{0}\\ y=0.5sin\left(\theta \right)+y_{0}\\ z=0.5\times v+z_{0} \end{array}\right. \end{array}$$

Here, ($x_{0}$, $y_{0}$, $z_{0}$) denotes the position of the odor source, and we set 0≤θ≤2π, 0≤v≤1. Ventilation was performed for at least 5 min in each experiment to evacuate the ethanol saturation in the experimental field. Ten trials were conducted for each odor source height. The pocket-sized quadcopter system was evaluated based on the search success rate and the time taken for the search to succeed.

### 6.2. Results

**Figure 17:**
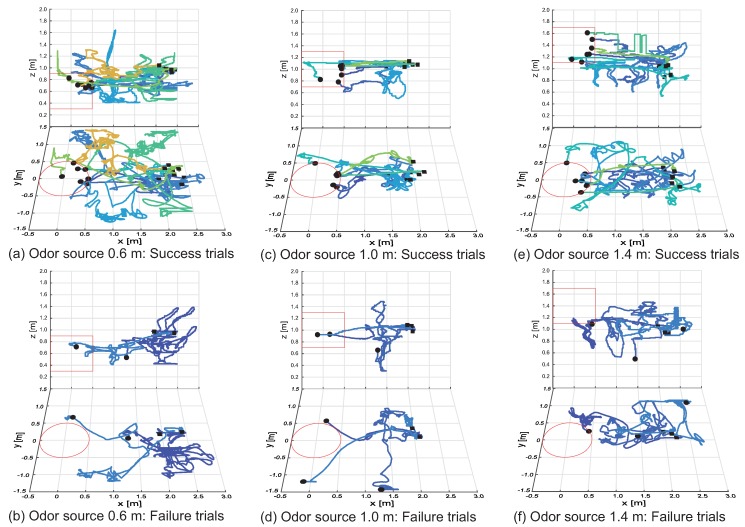
Migration pathway ratio map: subfigure (**a**) represents a success trial, and subfigure (**b**) represents a failure trial.

Figure 17 shows the results of search trajectories for the three-dimensional CPT experiments. (a–f) in Figure 17 represent trajectories for odor source heights of 0.6, 1.0 and 1.4 m, respectively. Moreover, (a–f) in Figure 17 represent trajectories for successful search and failure, respectively. We indicate the start and end position of CPT using “■” and “•”, respectively. In addition, the red line in Figure 17 indicates the goal area, while the different colors of the trajectory lines indicate each experimental trial. As shown in Figure 17c, the quadcopter reaches the goal area with few transitions to the loop state under conditions where the initial altitude of the quadcopter and the altitude of the odor source are the same. Conversely, as shown in Figure 17a,e, when the altitude of the odor source is different from the initial altitude of the quadcopter, we observed that the quadcopter transitions to the loop state, and the odor is searched in three dimensions. Focusing on the failure trajectory of Figure 17b,d,f, there are many trials in which the quadcopter strayed out of the odor distribution in the windward and crosswind directions. However, drawing attention to the height direction, the height of the search end position is almost the same as the altitude of the odor source; therefore, we found that the extension of the silkworm moth-inspired algorithm to three dimensions works effectively.

Next, we describe the results of the success rate and search time (shown in Table 3). The success rate was over 70% at any height condition of the odor source (see Supplemental Video S2). The shortest search time was achieved at an odor source height of 1.0 m, where the height of the odor source and the initial altitude of the quadcopter was the same. Moreover, when the height of the odor source is higher than the initial altitude of the quadcopter, the odor source was localized faster than when the odor source was lower than the initial altitude of the quadcopter. This is because we used ethanol, which is heavier than air, as the odor source. In other words, if the altitude of the odor source is low, the ethanol stays near the ground, and the quadcopter cannot obtain much odor information, which possibly causes the quadcopter to take a longer time in its search.

The reason that the search time is long or the search fails is because the radius of curvature of the search behavior (“zig-zag” and “loop” states) does not change. This result is the same as the result of the CPT experiment performed by Ando et al. in which silkworm moths were used [48]. Thus, the disadvantage of the silkworm moth CPT algorithm is that the possibility of failure of the search process increases if the quadcopter falls into a place where it cannot detect the chemical immediately. Therefore, to improve the search performance further, it is necessary to introduce an algorithm that searches in a wide range such that the chemical can be detected again when it is in a range in which it was otherwise impossible to detect [49,50].

These results imply that, although further development is necessary to realize three-dimensional CPT in unknown and cluttered environments, the intake system proposed in this paper enables a pocket-sized quadcopter to perform three-dimensional CPT.

## 7. Conclusions

In this study, we designed and verified an intake system for CPT using the wake of a pocket-sized quadcopter. The appropriate intake of air plays an important role in performing odor source searches. Therefore, we proposed an intake system that uses airflow produced by the quadcopter itself, because its low payload limitation does not allow room for the installation of additional actuators. Hence, we visualized the airflow around the quadcopter and placed the sensors at an appropriate location.

In addition, we employed a search algorithm based on the silkworm moth. The algorithm enables the quadcopter to perform an odor source search following a simple behavior pattern. However, we extended the algorithm to three dimensions because the silkworm moth performs CPT on a two-dimensional plane.

In the directivity performance verification experiment, the intake system showed good directivity, and this method proved effective for the pocket-sized quadcopter to realize CPT. Hence, we succeeded in giving odor directivity to the pocket-sized quadcopter.

Then, we conducted three-dimensional CPT experiments to evaluate the intake system. Even at different altitudes from the odor source, the quadcopter succeeded in searching for the source, with a success rate of 70% or higher. These results imply that, although further development is necessary so as to realize three-dimensional CPT in unknown and cluttered environments, the intake system proposed in this paper enables a pocket-sized quadcopter to perform three-dimensional CPT.

In this study, we focused on the silkworm moth CPT algorithm and extended it to three dimensions; therefore, the quadcopter had difficulties in discerning an odor distribution. In the future, we will combine surge-spiral or surge-casting algorithms [21], exploration plume-finding [50] and plume recontacting strategies [49] to improve search performance.

There are two possible ways to further improve the search success rate. The first method involves actively collecting odor information by moving in a wide range while searching for chemical cues. The second method involves searching for the odor source in cooperation with multiple quadcopters. The search performance can be increased by introducing multiple low-cost quadcopters and performing a parallel autonomous search, as opposed to using a high-performance UAV [51]. A Bluetooth beacon [52,53] can be used to communicate between the quadcopters locally.

Furthermore, in future work, we will implement obstacle avoidance by using a laser-ranging sensor (lightweight, only 0.5 g) that uses time-of-flight technology. In addition, we will focus on mixing the CPT algorithm with SLAM or other navigation algorithms (e.g., gas distribution mapping [54,55]) to achieve fully-autonomous navigation in disaster areas. Furthermore, we will tackle the problem of the quadcopter activity time (flight time) by extending the system to an aerial-ground robotic system, as reported in the literature [56].

Additionally, we may improve search accuracy by using different quadcopter sizes according to the size of the search space; the small drone is used indoors, and the large drone is for outdoors. Finally, we will extend our design to a system applicable for rescue missions by integrating elemental technologies such as sensing mechanisms, navigation algorithms, etc.

## Figures and Tables

**Table 1:** Definition of a quadcopter class [23].

Indoor	Classification	Frame Diameter (m)	Example
◯	Pocket size	≤0.2	Parrot Airborne
◯	Small size	≤0.6	Clearpath Hummingbird
△	Middle size	≤1.0	Airrobot AR100-B
×	Large size	>1.0	DJI Matrice 100

**Table 2:** Estimation result of the coefficients of the ARX inverse model.

$a_{1}$	$a_{2}$	$b_{0}$	$b_{1}$
−0.981	0.0165	0.283	−0.271

**Table 3:** Results of the three-dimensional CPT experiment. The search time is expressed in terms of the mean and standard deviation.

Source Height (m)	Search Success Rate (%)	Search Time (s)
0.6	80	74.7 ± 44.5
1.0	80	29.5 ± 22.3
1.4	70	56.8 ± 31.8

## Supplementary Materials

The following is available online at https://sshigaki.jimdo.com/research/: Video S1a: Free flight in particles for PIV; Video S1b: PIV analysis during the free flight; Video S2: CPT experiment video.

## Abbreviations

The following abbreviations are used in this manuscript:

CPT	Chemical plume tracing
ARX	Auto-regressive with an exogenous input
PIV	Particle image velocimetry

## Appendix A

We obtained the particle-tracking performance by calculating a Stokes number, St [57]. The Stokes number was derived by calculating the time constant of the equation of motion of the particle. Equation (A1) represents the equation of motion of the particles introduced into the flow field [58].

(A1) $$\begin{array}{c} \frac{\rho_{p}d_{p}^{2}}{18\mu_{f}}\frac{du_{p}}{dt}=u_{f}-u_{p} \end{array}$$

Here, $\mu_{f}$ (Pa·s), $u_{p}$ (m/s), $u_{f}$ (m/s), $\rho_{p}$ (kg/m^3^) and $d_{p}$ (m) represent the viscosity coefficient, particle velocity, velocity of measurement object, particle density and particle size, respectively. To measure motion accurately in the flow field in which the fluid accelerates, it is necessary for PIV particles to undergo the same acceleration motion. At this time, Equation (A1) can be expressed as shown in Equation (A2).

(A2) $$\begin{array}{c} |u_{f}-u_{p}|=\tau \left|\frac{du_{f}}{dt}\right|=\tau \left|\frac{du_{p}}{dt}\right| \end{array}$$

Equation (A2) expresses the time constant, τ.

(A3) $$\begin{array}{c} \tau =\frac{\rho_{p}d_{p}^{2}}{18\mu_{f}} \end{array}$$

We calculate the Stokes number by multiplying the time constant with the space-time scale, as shown in Equation (A4).

(A4) $$\begin{array}{c} St=\tau \frac{U}{L} \end{array}$$

Here, *U* and *L* represent the fluid velocity of the flow well away from the obstacle and the characteristic dimension of the obstacle, respectively. A decrease in the value of the Stokes number improves the tracking performance [57].

The PIV parameters used in this study are $\rho_{p}$ = 1.04 kg/m^3^, $d_{p}$ = 10.0 µm, $\mu_{f}$ = 1.82 × 10^−5^ Pa·s, *U* = 1 m/s and *L* = 0.15 m. Therefore, the Stokes number corresponds to St = 2.1 µ, and the 10-µm particle exhibits sufficient tracking performance.
